# University Teachers' Teaching Style and Their Students' Agentic Engagement in EFL Learning in China: A Self-Determination Theory and Achievement Goal Theory Integrated Perspective

**DOI:** 10.3389/fpsyg.2021.704269

**Published:** 2021-06-10

**Authors:** Anne Li Jiang, Lawrence Jun Zhang

**Affiliations:** ^1^School of Foreign Languages, Northeast Normal University, Changchun, China; ^2^Faculty of Education and Social Work, University of Auckland, Auckland, New Zealand

**Keywords:** teachers' teaching style, self-determination theory, achievement goal theory, achievement goals, agentic engagement

## Abstract

As a relatively new dimension of student engagement, agentic engagement has received growing research interest in recent years, as it not only predicts academic achievement and other positive outcomes, but also benefits reciprocal teacher-student relations. In the educational context, teachers' teaching style exerts a crucial impact on students' engagement. However, research on how perceived teachers' teaching style influences students' agentic engagement is inconclusive. To address this lacuna, this study, taking an integrated perspective that draws on Self-determination Theory and Achievement Goal Theory, investigated the relationship of three types of teaching style (i.e., perceived autonomy support, social relatedness, and controlling) to university students' agentic engagement in EFL learning in China, especially through the mediation of mastery-approach goals and performance approach goals. Structural equation modeling showed that perceived autonomy support positively predicted agentic engagement through the mediation of mastery-approach goals, whereas perceived controlling negatively predicted agentic engagement through the mediation of performance-approach goals. Comparatively, the relationship of perceived social relatedness to agentic engagement was fully mediated by both mastery-approach and performance-approach goals. After discussing these results, practical implications as well as suggestions for future studies were given.

## Introduction

Over the past three decades, academic engagement has been a key and heavily researched topic for psychologists and educational researchers (see Boekaerts, 2016), even in the fields of second or foreign language education (e.g., Sun and Zhang, 2020; Zhang and Zhang, 2020; Jiang and Zhang, 2021). It is well-established that high levels of engagement are reliable forerunners of students' learning satisfaction, positive academic outcomes, perseverance, and school completion rates (Wang and Holcombe, 2010; Uçar and Sungur, 2017). Agentic engagement, on top of cognitive, emotional, and behavior engagement, is a relatively new dimension (Reeve and Tseng, 2011; Reeve, 2013). Rather than merely representing students' reaction to the learning conditions and to teachers' instruction behaviors like the other three types of engagement, agentic engagement also emanates students' agency to proactively personalize and contribute to knowledge construction and the on-going flow of instruction (Reeve and Tseng, 2011). In recent years, this concept has received growing research interest as it not only predicts academic achievement and other positive outcomes, but also benefits reciprocal teacher-student relations. However, compared with the other three types of engagements, empirical research on the determinants and correlates of students' agentic engagement is inconclusive.

As a key component for understanding teaching and learning in the educational context, teachers' interpersonal teaching style exerts a crucial impact on students' engagement. Informed by Self-determination Theory (SDT), a bulk of studies have investigated the effects of teachers' teaching style on students' need satisfaction, motivation, emotion, and learning outcomes (e.g., Vansteenkiste et al., 2004; Jang et al., 2012; Haerens et al., 2015). However, research on how it influences students' agentic engagement is still in its infancy. The few studies that have examined this issue are grounded in SDT and focus primarily on the bearing of autonomy-supportive teaching on students' motivation or needs satisfaction, which in turn, affects their agentic engagement (e.g., Jang et al., 2012; Cheon and Reeve, 2013; Reeve and Shin, 2020). Other types of teaching style still await investigation. Moreover, students' goal pursuit, which is the focus of Achievement Goal Theory (AGT), another important motivational theory complementary to SDT, is also an important motivational predictor of students' engagement. Actually, there is a growing call for these two theories to be neatly integrated in exploring students' motivated behavior (e.g., Ciani et al., 2011; Soini et al., 2014). To this effect, we attempt to take an integrated perspective drawing on both SDT and AGT to entangle the relationship between teachers' teaching style, students' achievement goals, and agentic engagement in EFL learning.

Given that teachers' teaching style includes multiple aspects (see e.g., Reeve, 2013; Soini et al., 2014; Wang and Zhang, 2021), the present study focuses specifically on three of them: autonomy support, social relatedness, and controlling. For one thing, the relationship between autonomy-supportive teaching and agentic engagement has been studied only by a limited number of studies, hence the need to enrich this line of research. Meanwhile, we assume that, as the opposite of autonomy support, controlling should influence agentic engagement in a different pathway which is worth exploration. For another, the rationale to include the aspect of social relatedness lies in our intention to extend previous research on teachers' teaching style and cater to culture-related concern. Regarding that agency may be culturally-distinctive (Markus and Kitayama, 2003), we are curious about the way that the agentic engagement of Chinese university students, who have grown up in a collectivistically oriented culture, can be affected by teachers' teaching that promotes social relatedness. Moreover, this study is set in the context of teaching English-as-a-foreign-language (EFL) to complement previous relevant studies that were mostly set in disciplinary areas such as physical education or mathematics. It is believed that the present study will be conducive to a more comprehensive understanding of how teachers' teaching style influences students' agentic engagement both theoretically and empirically.

## Literatue Review

### Self-Determination Theory and Achievement Goal Theory: A Necessity to Integrate

Self-determination Theory (SDT, Deci and Ryan, 1985) has been established as a heuristic theoretical framework to study peoples' motivated behavior in various contexts including school settings (Ryan and Deci, 2020). It posits that people have three innate psychological needs: autonomy (i.e., experiencing a sense of volitional and psychological freedom), relatedness (i.e., experiencing closeness and mutuality in interpersonal relationships), and competence (i.e., experiencing personal effectiveness). The nurturing of these needs is positively associated with high-quality motivation, engagement, well-being, and adaptive functioning. In contrast, thwarting these needs would undermine individuals' psychological well-being and generate negative affect, amotivation, and maladaptive functioning (e.g., Vansteenkiste et al., 2004; Cheon and Reeve, 2015).

SDT assumes that individuals' motivated behaviors vary contingent on the extent to which they are experienced as autonomous vs. controlled. Following Deci and Ryan (1985), the prototype of autonomy (or self-determination) is intrinsic motivation, and intrinsically motivated people engage in an activity for its own sake rather than for external pressure of outcomes separated from the activity *per se*, as in the case of extrinsically motivated behavior. In other words, SDT differentiates between autonomously motivated behaviors endorsed by volition and psychological freedom and controlled behaviors executed with a sense of pressure or obligation. From an SDT view, motivation exists along a continuum of relative autonomy. Extrinsic motivation can become intrinsic motivation through the process of internalizing the initially external regulation for behaviors. External regulation, such as rewards, punishment, and deadlines, is the least autonomous form of extrinsic motivation. When partially internalized by the individual, external regulation becomes introjected regulation. When introjected regulation is further internalized by the individual who identifies with the value of an activity, it becomes identified regulation. External regulation and introjected regulation are categorized as controlled motivation, whereas intrinsic motivation and the identified regulation are regarded as autonomous or self-determined motivation (Ryan and Deci, 2020).

Apart from SDT, Achievement Goal Theory (AGT) provides an alternative and complementary theoretical perspective on individuals' motivated behavior (Wolters, 2004). AGT proposes that in an achievement-related situation like an educational context, a person's motivation and achievement-related behaviors is affected by his/her goal orientations, which refer to how success is perceived and competence evaluated. Traditionally, two goal types have been primarily emphasized, namely mastery goals, and performance goals. The former focus on the development of competence through task mastery, whereas the latter highlight the demonstration of competence in comparison with others.

To date, the mastery and performance dichotomy has been extended to a 2 × 2 conceptualization including a distinction between approach goals and avoidance goals, representing the distinction between the need for success and fear of failure (Elliot and McGregor, 2001). Students adopting a mastery-approach goal orientation prioritize learning as much as possible and increasing their level of competence. Students with a mastery-avoidance goal orientation tend to work to avoid lack of mastery of knowledge. Students holding a performance-approach goal orientation are inclined to demonstrate their ability relative to others. Finally students with a performance-avoidance goal orientation would try to avoid looking incompetent or less able than their peers (Wolters, 2004).

Both SDT and AGT see learning environment as important affordance for students' engagement. A theoretical proposition of SDT is that contextual features, especially teachers' interpersonal teaching style, can influence students' motivation and engagement by nurturing vs. thwarting three basic psychological needs (see Wang and Holcombe, 2010). In a similar vein, AGT also endorsed the important role of teachers' teaching style. AGT assumes that students' engagement is influenced by goal pursuits which can be directed by classroom goal structures created largely by the teacher. In most cases, when students perceive the predominant instructional and evaluation practices and strategies as mastery goal oriented in the classroom contexts, they tend to demonstrate higher levels of engagement, Conversely, a performance goal structure is often found negatively correlated with students' school engagement (see Meece et al., 2006; Diseth and Samdal, 2015). However, an obvious distinction between the two theories is that AGT operates mainly with perceived competence, which is considered the main motive of human behavior, whereas in addition to the perception of competence, SDT also involves autonomy and social relatedness as important predictors for motivation and behavior. Yet SDT does not treat competence as multidimensional as AGT.

Considering the connection and distinction between the two theories, it is necessary to take an integrated perspective in investigating the influence of teachers' teaching style on students' engagement.

### Teachers' Interpersonal Teaching Style

Informed by SDT, teachers' interpersonal teaching style can differ in the extent to which it supports students' basic psychological needs for autonomy, relatedness, and competence. To date, a multitude of research on teachers' interpersonal teaching style in the SDT tradition has focused on the effects of autonomy support teaching and controlling teaching on students' need satisfaction, motivation, emotion, and learning outcomes (e.g., Jang et al., 2012; Cheon and Reeve, 2013; Haerens et al., 2015). Autonomy support is understood as a cluster of teachers' instructional behaviors to provide students with an environment and a teacher-student relationship that can support students' needs for autonomy. Autonomy-supportive teachers adopt various strategies to frame the lesson within a context of intrinsic goal pursuits and autonomy support, and provide explanatory rationales when requesting students to engage in less interesting activities. Research has found that autonomy-supportive teaching can promote students' identified regulation of the activity and their internalization of motivation, and consequently has positive effects on students' needs satisfaction, high-quality motivation, and engagement (e.g., Jang et al., 2012; Jin et al., 2020).

Controlling teaching, on the contrary, involves utilizing intrusive behaviors to pressure students to think, feel, or behave in a specific way prescribed by the teacher (Reeve, 2009). A controlling teaching style can manifest primarily in two ways: direct (or external) control, and indirect (or internal) control. Teachers demonstrating direct control through using overt external compulsions to motivate students to act, including the imposition of punishments, rewards, deadlines, and verbal commands. Indirect control involves teachers using more covert tactics to motivate students, such as arousing students' feelings of guilt, shame, and anxiety, threatening to withdraw attention, and cultivating perfectionist standards (Soenens and Vansteenkiste, 2010). Both direct and indirect controlling would induce controlled regulation on the part of the students (Reeve, 2009). Such low-quality motivation, as shown in prior research will undermine students' full range of engagement (e.g., Reeve, 2009; Haerens et al., 2015).

Compared with autonomy-supportive teaching, controlling teaching is relatively less investigated in its own right (Haerens et al., 2015). In some studies, both teaching styles were assessed as dual processes informed by SDT (e.g., Jang et al., 2020), which have yielded particularly useful insights showing that different teaching styles influence students' behavior in different ways. As Haerens et al. (2015) argued, it is necessary to consider the contribution of both styles, for the absence of one does not necessarily imply the presence of the other. Additionally, they may show differential associations with different types of students' motivation and behavior.

However, compared with autonomy support and controlling teaching, other types of teaching style, such as relatedness-supportive teaching, structuring teaching, task-involving support teaching, and ego-involving support teaching, among others (see Soini et al., 2014; Ryan and Deci, 2017), are relatively rarely researched in terms how they influence students' motivation and learning behavior. It is necessary to extend previous literature by examining these other types of teaching style.

### Students' Agentic Engagement

Students' learning engagement refers to “the quality of a student's connection or involvement with the endeavor of schooling and hence with the people, activities, goals, values and place that compose it” (Skinner et al., 2009, p. 494). Most engagement theorists conceptualize it as a multidimensional construct, which is generally assumed to include three interrelated and well-studied dimensions: emotional engagement, cognitive engagement, and behavioral engagement. Emotional engagement represents students' positive or negative affective states when the students are doing the learning activities. Cognitive engagement concerns psychological investment and being strategic or self-regulating in learning. Behavioral engagement represents students' observable behaviors showing on-task effort and commitment in learning (Skinner et al., 2009).

The concept of agentic engagement is initially proposed by Reeve and Tseng (2011) and defined as “students' constructive contribution into the flow of the instruction they receive” (p. 258). Similar to the above three types of engagement, agentic engagement is also a student-initiated pathway to academic progress, but it is a uniquely proactive and transactional type of engagement. It is proactive in the sense that agentively engaged students take action before a teacher-initiated or directed activity begins. It is transactional because students will agentively work for a more motivationally supportive learning environment through, for instance, expressing their preferences and interests, asking questions, and expressing their needs (Reeve, 2013). In addition, students' agentic engagement can lead to reciprocal causation in teacher-student interactions, which means the teacher's behavior and the students' behavior will mutually influence each other during instruction. Overall, agentic engagement is a crucial element to improve students' learning and render the learning environment more motivationally supportive (Reeve and Shin, 2020).

Compared with behavioral, emotional and cognitive engagements, there is a relative paucity of empirical research regarding agentic engagement. Reeve and Tseng (2011) made a comparison of the four dimensions of engagement and found agentic engagement predicted independent variance in the participants' achievement. Employing a 3-wave longitudinal design, Reeve (2013) validated a positive association between agentic engagement and autonomous motivation. His research also found that agentic engagement contributes to greater achievement and a supportive learning environment, thus indicating its reciprocal potential to change the learning context. The potential for agentic engagement to predict teachers' autonomy-supportive motivating style is empirically validated by a few longitudinal studies (e.g., Reeve, 2013, Matos et al., 2018; Reeve and Shin, 2020). A limited number of studies have suggested teachers' autonomy-supportive teaching style as a predictor of students' agentic engagement (e.g., Jang et al., 2012; Cheon and Reeve, 2013; Reeve and Shin, 2020). Most of this line of research is informed by SDT, thus taking either motivation (i.e., intrinsic motivation, external motivation, amotivation) or need satisfaction and frustration as the mediators in the relationship of autonomy-supportive teaching to students' agentic engagement. More empirical research with new theoretical perspectives and affecting factors is imperative.

### Master-Approach and Performance-Approach Goals as Potential Mediators

In the first section of theoretical elaboration, it can be seen that both SDT and AGT are useful in understanding students' motivation, its antecedents, and ensuing behavioral patterns like engagement. A body of research has examined the association between AGT- and SDT-based concepts and ideas. It is indicated that motivation and the satisfaction of basic psychological needs can act as the antecedents of people's goal adoption (see Ciani et al., 2011). For instance, master-approach goals were found to be positively related to intrinsic and identified motivation, but negatively to external motivation, whereas performance goals were either unrelated or negatively related to intrinsic motivation (Barkoukis et al., 2007). In a similar vein, studies examining autonomous and controlling reasons underlying the pursuit of mastery and performance goals suggest that when students act out of autonomous reasons, i.e., reasons that are consistent with intrinsic motivation or identified regulation in nature, they are more likely to pursue mastery-approach goals. Conversely, when students act out of controlled reasons, i.e., reasons aligned with introjected regulation and external regulation (extrinsic motivation), they are more likely to pursue performance-approach goals (e.g., Gaudreau, 2012; Benita et al., 2014; Gillet et al., 2015). Moreover, basic needs satisfaction can be the source of intrinsic motivation, which subsequently predicts mastery-approach goals (Ciani et al., 2011).

Informed by SDT and AGT, teachers' interpersonal teaching style is an important motivational element in the learning environment that will affect students' motivation and achievement goal orientation, which can subsequently affect engagement. Meanwhile, different types of teaching style may work on students' motivated behavior in distinctive ways, as they may cause different reasons for motivated action. These reasons can promote either mastery-approach goals or performance-approach goals, which will subsequently affect students' behavior. Therefore, it is expected that mastery-approach and performance-approach goals may function as mediators between students' perceived teaching style and their agentic engagement.

### Hypotheses and Question

**Figure 1:**
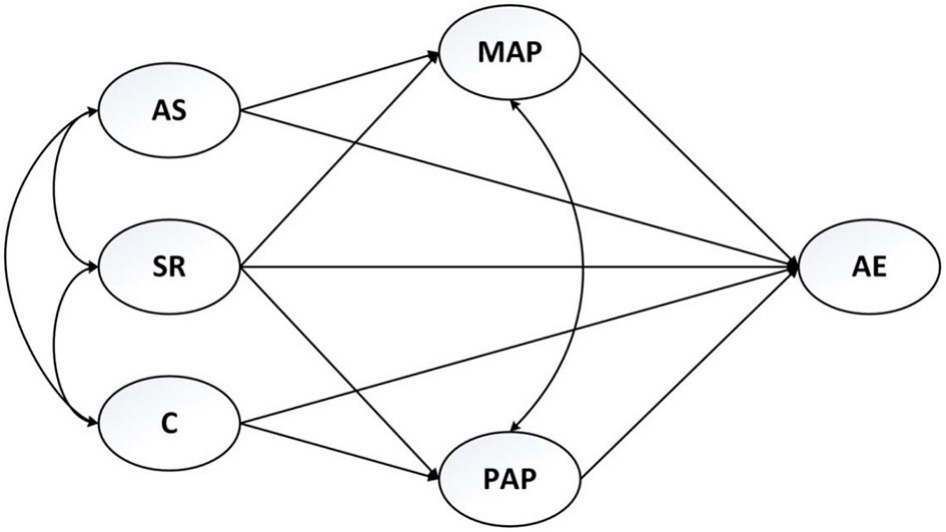
The research model. AS, perceived autonomy support; SR, perceived social relatedness; C, perceived controlling; MAP, mastery-approach goals; PAP, performance-approach goals; AE, agentic engagement.

Taking an SDT and AGT integrated perspective, the present study aims to entangle the relationship among university students' perceived autonomy support, controlling, and social relatedness (i.e., students' perception of three types of teaching style), mastery-approach goals and performance approach goals, and their agentic engagement in EFL learning in China. Considering that different types of teaching style can influence engagement through distinct pathways as informed by the above literature review, the present study makes two hypotheses (i.e., H1 and H2) regarding how perceived autonomy support and controlling will influence students' agentic engagement. However, as there is a lack of empirical literature on the how perceived relatedness will influence agentic engagement, we raise an exploratory question, which is to be answered through testing the hypothetical pathways in the researched model. Figure 1 shows the researched model demonstrating the two hypotheses and the hypothetical pathways to answer the question.

H1: Students' master-approach goals will mediate the relationship of perceived teacher autonomy support to their agentic engagement in EFL learning.
H2: Students' performance-approach goals will mediate the relationship of perceived teachers' controlling teaching to their agentic engagement in EFL learning.
Question: How does perceived social relatedness relate to agentic engagement in EFL learning? Is their relationship mediated by mastery-approach goals and/or performance-approach goals?

## Method

### Context and Participants

English is a mainstream subject in the curriculum of secondary education and a common core course for most undergraduates in universities in China. In secondary education, the primary objective of English teaching is to set a solid language foundation for students, and as a subject, English takes up a considerable proportion of the total score in important high-stake exams, such as the Gaokao, which is also known as the National Higher Education Entrance/Matriculation Examination. In university, the objective of English teaching, while continuing and heightening that of secondary education, is to cultivate students' cross-cultural communicative competence, and to promote their language proficiency for academic or professional purposes (Ministry of Education of the People's Republic of China, 2020). In most universities, English for general purposes and English for academic purposes are among the major English courses available to undergraduate students (non-English majors). In terms of pedagogy, in the past two decades, English teaching has gradually shifted its focus from teacher-centered grammar teaching to student-centered communicative-competence development in both secondary and higher education.

In the present study, we targeted student participants who are non-English majors admitted to universities after taking the Gaokao and have been attending an English course for at least half a semester, because they can represent the English learning experience of most Chinese university EFL learners.

### Instrument

We assessed students' perception of their EFL teachers' autonomy support, controlling and social relatedness using *Perceived Teachers' Teaching Style Scale* (PTTSS), which was adapted first through consulting the Motivational Climate in Physical Education Scale (Soini et al., 2014). This scale contains four factors assessing students' perceptions of autonomy support, social relatedness, mastery goal motivational climate, and performance goal motivational climate that are afforded by the learning environment, especially by teachers' interpersonal teaching style. The autonomy support and social relatedness scales were selected. We kept but rephrased the original items by adding a stem or elaboration to some of them. For instance, the original item “It is important for the students to try to improve their own skills” was rephrased as “Our English teacher makes me aware that it is important to try to improve my own skills.” Then, to assess teachers' controlling teaching style, we referred to Reeve's (2016) description of teachers' controlling teaching behaviors, which compared to similar measures in some other research, are more close to the reality of university EFL teachers' practice. We adapted and included in 5 items (e.g., “Our English teacher does not understand our learning needs, objectives, or preference”). In this manner, the PTTSS ended up with three scales, i.e., autonomy-supportive teaching (with 5 items, Cronbach's α = 0.86), social relatedness motivating teaching (with 4 items, Cronbach's α = 0.80), and controlling teaching (with 5 items, Cronbach's α = 0.82).

To assess students' mastery-approach goals and performance-approach goals, we adopted the corresponding items from Elliot and McGregor's (2001) Achievement Goal Questionnaire. Each dimension has three items slighted revised to be contextualized (i.e., the words “In our English class” was added initiate each item, such as “In our English class, it is important for me to do better than other students”). The Cronbach's α for mastery-approach goals was 0.82, and 0.83 for performance-approach goals.

Students' agentic engagement in EFL learning was assessed using the corresponding items from Reeve's (2013) work. The original items were slightly contextualized by adding words like “In our English class.” This scale contains 5 items and demonstrated good reliability in the present study (Cronbach's α = 0.93). See Appendix for the details of the whole questionnaire.

### Data Collection

With the help of 9 university EFL teacher colleagues, we distributed an online questionnaire that was available on Wenjuanxing, an online crowdsourcing platform, to potential participants in the middle of the winter semester of 2020. Participation was voluntary and responses were confidential and anonymous. Finally, 632 undergraduate students (71.5% females, 28.5% males; 77% Year 1; 22% Year 2) from different universities (73% national universities; 27% provincial universities) in mainland China (86% northern China, 14% eastern and southern China) completed the questionnaire.

### Data Analysis

SPSS 22 was utilized to conduct descriptive analysis, bivariate correlational tests, and reliability tests of the questionnaire. Confirmatory factor analysis (CFA) using AMOS 23 was then performed to validate the instruments. During the initial process of establishing the measurement model, the composite reliability (CR), the average variance extracted (AVE), and the square roots of average variance extracted were calculated to assess the reliability and validity of each variable. Afterwards, structural equation modeling (SEM) using AMOS 23 was adopted to confirm the measurement model and test the model fit of the structural model. The indices used to test the fit of the models include the χ^2^ statistics, Tucker-Lewis index (TLI ≥0.95), comparative fit index (CFI ≥0.95), root-mean-square error of approximation (RMSEA ≤ 0.06), and standardized root mean square residual (SRMR ≤ 0.08, Hu and Bentler, 1999). It should be noted that the χ^2^ statistics is sensitive to sample size, so the above alternative indices were primarily consulted for data-model fit evaluation, and a value of 5 or less for the ratio between chi-square and degree of freedom (χ^2^/*df* ≤ 5) was adopted following Schumacker and Lomax (2004).

## Results

### Preliminary Analyses

We first intended to check the inter-relationships among the variables of perceived autonomy support (AS), social relatedness (SR), controlling (C), mastery-approach goals (MAP), performance-approach goals (PAP), and students' agentic engagement (AE). Our analysis of the questionnaire data produced interesting results. Table 1 presents the correlational coefficients, means, and standard deviations of these focal variables. Based on the results, perceptions of all three types of teaching styles (i.e., autonomy support, social relatedness, and controlling) were positively correlated with the hypothesized mediators of goal orientations (i.e., mastery-approach goals and performance-approach goals), as well as the outcome of agentic engagement, except for the only non-significance between perceived controlling and mastery-approach goals.

**Table 1:** Descriptive statistics and bivariate correlations among the scales (*n* = 632).

**Measurement variables**	**AS**	**SR**	**C**	**PAP**	**MAP**	**AE**
AS	1					
SR	0.67^**^	1				
C	0.24^**^	0.4	1			
PAP	0.23^**^	0.15^**^	0.26^**^	1		
MAP	0.23^**^	0.34^**^	−0.02	0.3^**^	1	
AE	0.54^**^	0.43^**^	0.1^**^	0.31^**^	0.28^**^	1
Mean	4.01	4.45	2.71	3.41	4.17	3.61
SD	0.80	0.60	1.02	0.89	0.68	0.82
Skewness	−0.39	−0.78	0.74	−0.24	−0.43	0.04
Kurtosis	−0.46	−0.40	0.23	0.15	−0.29	−0.32

** *p < 0.01 (two-tailed)*.

### Measurement Model and Common Method Variance

Prior to the structural model analyses, we assessed the measurement model including the six latent variables (i.e., perceived autonomy support, perceived social relatedness, perceived controlling, mastery-approach goals, performance-approach goals, and agentic engagement) by means of confirmative factor analyses (CFA). The results indicated an acceptable model-data fit: χ^2^/*df* = 2.59; CFI = 0.95; TLI = 0.94; RMSEA = 0.05; SRMR = 0.06. In addition, all factor loadings ranged from 0.60 to 0.92 at the significant level of *P* < 0.001 (see Table 2).

**Table 2:** CFA results of the measurement model (*n* = 632).

**Factors**	**Factor loading**	**CR**	**AVE**	**Square roots of AVE**
Autonomy support (AS)		0.86	0.55	0.74
AS1	0.73			
AS2	0.71			
AS3	0.78			
AS4	0.75			
AS5	0.74			
Social relatedness (SR)		0.79	0.49	0.70
SR1	0.66			
SR2	0.61			
SR3	0.68			
SR4	0.82			
Controlling (C)		0.82	0.49	0.70
C1	0.77			
C2	0.78			
C3	0.63			
C4	0.60			
C5	0.69			
Mastery-approach goal (MAP)		0.82	0.61	0.78
MAP 1	0.79			
MAP 2	0.79			
MAP 3	0.76			
Performance-approach goal (PAP)		0.84	0.64	0.80
PAP1	0.81			
PAP2	0.90			
PAP3	0.68			
Agentic engagement (AE)		0.92	0.70	0.84
AE1	0.83			
AE2	0.82			
AE3	0.85			
AE4	0.85			
AE5	0.84			

As the data were collected from the self-reported measures, it is necessary to examine whether the common method bias would affect the results. Following the suggestions of Podsakoff et al. (2003), we conducted the single factor analysis with all 25 items forced to load on a single factor, which extracted only 36.35% of the total variance, a percentage well below the warning cut-off criterion of 50% (Cao et al., 2020). The results showed that common method bias may not be a problem for the present study.

### Structural Model Analyses

**Figure 2:**
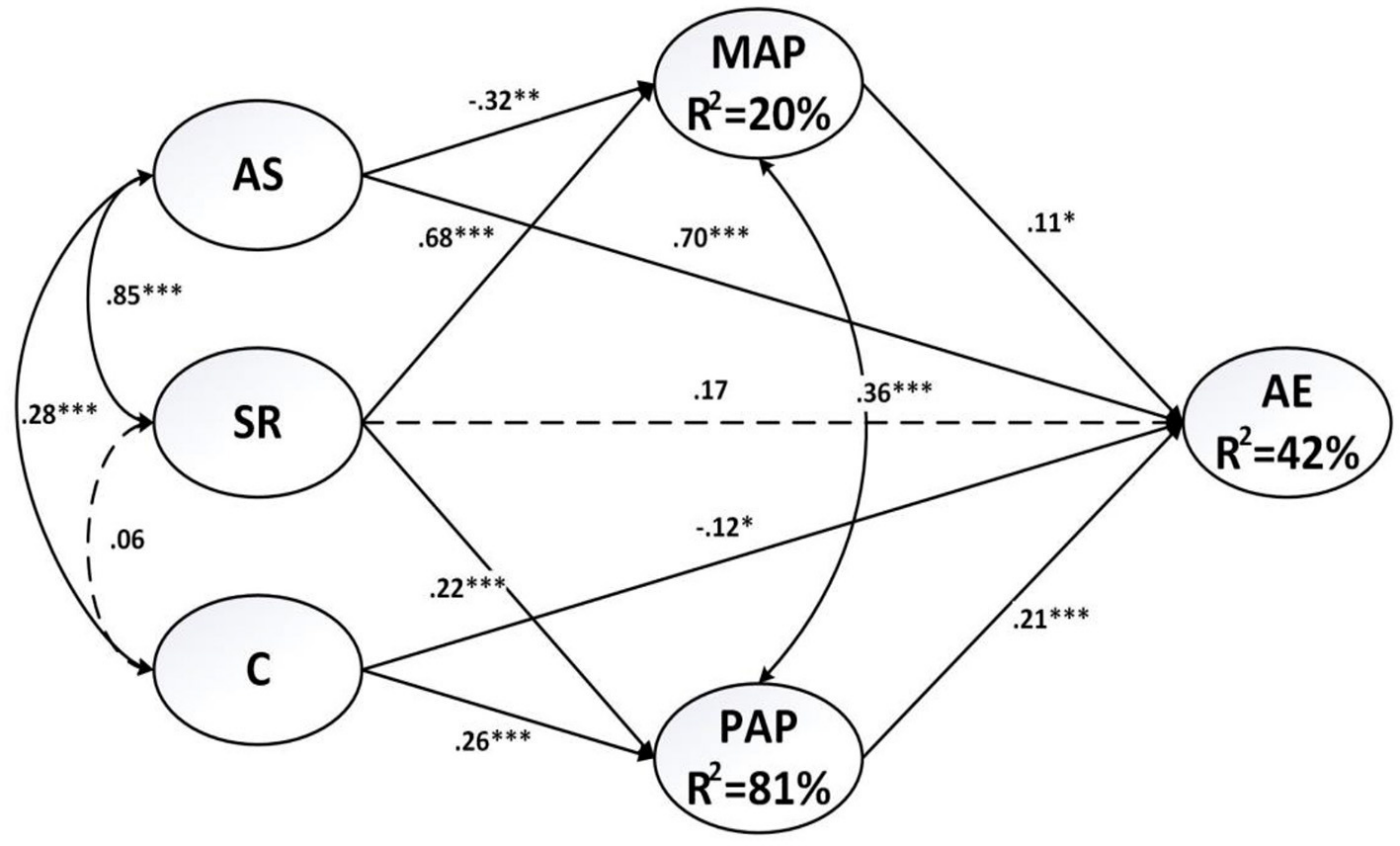
Results of testing the structural model. *p < 0.05, **p < 0.01, and ***p < 0.001. The solid lines indicate significant paths; the dotted lines indicate non-significant paths. AS, perceived autonomy support; SR, perceived social relatedness; C, perceived controlling; MAP, mastery-approach goals; PAP, performance-approach goals; AE, agentic engagement.

After confirming the measurement model, we then assessed the structural model which received an acceptable model-data fit as well: χ^2^/*df* = 2.59; CFI = 0.95; TLI = 0.94; RMSEA = 0.05; SRMR = 0.06. Figure 2 presents the standardized path coefficients. According to the results, perceived autonomy support negatively predicted mastery-approach goals (β = −0.32, *p* = 0.008) and positively predicted agentic engagement (β = 0.70, *p* < 0.001). Perceived social relatedness positively predicted mastery-approach goals (β = 0.68, *p* < 0.001) and performance-approach goals (β = 0.22, *p* < 0.001), but was non-significant for agentic engagement (β = 17, *p* = 0.187). The last type of teaching style, namely perceived controlling, positively predicted performance-approach goals (β = 0.26, *p* < 0.001), but negatively predicted agentic engagement (β = −0.12, *p* = 0.013). Finally, both mastery-approach goals (β = 0.11, *p* = 0.042) and performance-approach goals (β = 0.21, *p* < 0.001) positively predicted agentic engagement.

### Mediational Analyses

Mediating effects of mastery-approach goals and performance-approach goals were examined by means of the bootstrapping method in SEM. According to Shrout and Bolger (2002), when the 95% confidence intervals (CI) does not contain zero, we determine that the indirect effect can be significant.

The result revealed that AS had significant and indirect relationships with agentic engagement (95% CI [−0.135, −0.003]), and this relationship was mediated by mastery-approach goals. Thus, H1 was supported. It was also revealed that perceived controlling had a significant and indirect relationship with AE (95% CI [0.026, 0.092]), and this relationship was mediated by perceived-approach goals. Therefore, H2 was supported. Perceived social relatedness had a significant and indirect relationship with agentic engagement (95% CI [0.054, 0.247]), and this relationship was fully mediated by mastery-approach goals and performance approach goals, since the direct pathway between perceived social relatedness and agentic engagement was non-significant. Thus, the answer to the research question is that both mastery-approach and performance-approach goals fully mediated the relationship of perceived social relatedness to agentic engagement (see Figure 2).

## Discussion

Drawing on a combined perspective of SDT and AGT, we undertook the present study to investigate how Chinese university students' perceptions of three dimensions of teachers' interpersonal teaching style (i.e., autonomy-support, social relatedness, controlling) may play a role in their agentic engagement in EFL learning, specifically through the mediation of their master-approach and performance-approach goal orientations.

We first hypothesized that mastery-approach goals would mediate the relationship of perceived autonomy support and agentic engagement. The results supported this hypothesis in an interesting way. Convergent with previous studies (Jang et al., 2012; Cheon and Reeve, 2013), perceived autonomy support positively predicted agentic engagement in this study. However, the relationship between perceived autonomy support and mastery-approach goals was negative. So we guess that the significant positive predication of perceived autonomy support on agentic engagement might occur largely through the mediation of students' need satisfaction (Jang et al., 2012; Cheon and Reeve, 2013; Reeve, 2013; Reeve and Shin, 2020) and students' perceived autonomous reasons for action (Benita et al., 2014) from an SDT perspective. In the context of EFL teaching, take writing class for instance, various sources of feedback, either technology-assisted autonomatic feedback, teachers' feedback, or peers' feedback, which are intended to support students' autonomy, can promote students' agent engagement in teacher-student and student-student interactions, largely because these sources satisfy students' needs for competence, i.e., to improve their language proficiency (see Tian and Zhou, 2020). However, the result that autonomy support was negatively associated with master-approach goals is a bit beyond expectation, because theoretically as previous elaborated, teachers' autonomy supportive teaching contributes to building an autonomy supportive classroom goal structure, which in turn, should promote students' master-approach goal orientation (Meece et al., 2006). We reckon the conflicting result of the present study may be ascribed to two reasons. One is that the predicative power of autonomy supportive teaching on goal orientations may be influenced by some other motivational variables, like self-efficacy as suggested in Greene et al.'s study (Greene et al., 2004). The other reason is related to our curiosity that to what extent an autonomy supportive teaching style can gauge the goal orientations of students who are still new to such a style. The majority of the participants in this study were in their first year of university. Though the curriculum reform has long been endeavoring to transform Chinese schooling from teacher-centered education to student-centered learning characterized by active student engagement, and has achieved some positive outcomes (see Adams and Sargent, 2012), the long established exam-oriented middle education context headed by the Gaokao (i.e., National Higher Education Entrance Examination) may push students to learn for non-self-determined reasons (see Yu et al., 2016 for a systemic review). The highly competitive and controlling environment, and the emphases on out-performing peers in middle schools, might have deprived students of the chance to explore and form genuine identity, interests, self-values or definite direction for life before they enter university (Yu et al., 2016). Facing an autonomy-advocated teaching style more prevalent in higher education contexts, which might be in sheer contrast to their familiar controlling system in previous learning contexts, it is understandable that students may temporarily fail to develop goals for knowledge mastery and self-improvement. Or even worse, it could be the case that the more teachers give them autonomy, the more students would feel at a loss concerning their goal orientation.

In the second hypothesis, we assumed that perceived controlling would predict agentic engagement through the mediation of performance-approach goals. This hypothesis was supported by the results. First, informed by SDT, controlling teaching is prone to induce controlled regulation on the part of the students (Reeve, 2009). Subsequently, controlled regulation, or controlled reasons as it also called, tends to induce performance goals as theoretically explained in both SDT and AGT literature (e.g., Gaudreau, 2012; Benita et al., 2014; Gillet et al., 2015). Such a chain relationship is empirically validated by the significant positive prediction of perceived controlling on students' performance-approach goals in the present study. Moreover, the result that performance-approach goals positively and significantly predicated agentic engagement suggests that performance-approach goals can contribute to agentic engagement in certain educational context like EFL teaching and learning. Specifically, as elaborated in the above paragraph, the majority of the participants in this study had left behind a competition-oriented context pressured by the Gaokao not long ago. Even in university, there is still the competition for scholarship, GPA ranking or better job opportunities. In this situation, learning for higher grades and outperforming others may still be the major goal for some students. To achieve this goal may give them the incentive to work for a preferable learning environment conducive to achieving their goals. In this sense, the result supports the view that performance-approach goals can be beneficial and adaptive in the educational context (see Vansteenkiste et al., 2010; Ciani et al., 2011). Additionally, this result also extends previous research examining the influence of performance-approach goals on engagement, as a good deal of evidence has to do mainly with cognitive, behavioral, and emotional engagement but ignores agentic engagement (e.g., Greene et al., 2004; Wang and Holcombe, 2010; Uçar and Sungur, 2017; Sun and Zhang, 2020).

However, though perceived controlling positively predicted performance-approach goals which in turn positively predicted agentic engagement, perceived controlling was negatively associated with agentic engagement. A possible explanation is that for the participants in this study, the combined effect of perceived controlling and performance-approach goal is too strong for their agentic engagement. This is because perceived controlling teaching is closely related to need frustration and subsequent controlled motivation, amotivation, and maladaptive functioning (Vansteenkiste et al., 2004; Cheon and Reeve, 2015; Haerens et al., 2015). Performance-approach goals can be related to anxiety, disruptive behavior, and low retention of knowledge (Midgley et al., 2001). When such negative effects and outcomes aggregate, it is presumable that the participants' initiative to join force with the teacher to build a more preferable learning environment will be thwarted.

As for the research question, the results suggested both mastery-approach and performance-approach goals played a full mediating role in the relationship of perceived social relatedness to agentic engagement. This result can shed light on how relatedness acts as a precursor to achievement goals in a certain context. It also indicates that satisfying students' needs for relatedness is crucial in EFL teaching and learning, as it is in other disciplinary settings such as physical education (Vansteenkiste et al., 2010; Cheon and Reeve, 2013; Jang et al., 2020). Given that English teaching changed its focus from teacher-centered grammar teaching to student-centered communicative-competence development in China in the past two decades, task-based language teaching has been extensively advocated and applied in secondary and higher education sectors (Zheng and Borg, 2014; Xu and Fan, 2021). To complete tasks successfully requires students' frequent collaboration with peers (e.g., in the form of pair-work or group-work). When teachers' teaching promotes social relatedness in EFL learning, students' basic need for relatedness is satisfied, and their closeness with peers can make collaboration more enjoyable and effective. This can not only affords them with autonomous reasons for learning, and thus can stimulate their pursuit for mastery-approach goals, but also promotes their performance-approach goals. Both goal orientations are positive predictors of students' agentic engagement in EFL learning. Additionally, this result also supports the cross-cultural validity of SDT. Some cross-cultural researchers have argued that psychological need satisfaction proposed by SDT might not yield the same education benefits for Eastern collectivistic cultures as found in Western cultures (see Jang et al., 2009 for a review). However, our research, congruent with Jang et al.'s (2009) work, suggests that satisfied needs for social relatedness indeed produce positive learning experience for students as it can promote students' agentic engagement in China, another Eastern country of collectivistic cultural heritage.

The findings of this study might have practical implications. First, when trying to encourage students' agentic engagement through various teaching styles, teachers should take student's achievement goal orientation into consideration. While both mastery-approach goals and performance-approach goals can boost agentic engagement, not every teaching style can definitely influence these goals in all conditions. In order for autonomy-supportive teaching style to generate more positive outcomes, it is necessary for teachers to consider students' prior learning experience, especially their personal values, interests, and authentic identity related to learning. Moreover, controlling teaching is not always negative. Under certain conditions, it helps to enhance students' performance-approach goal pursuits, which may subsequently generate positive outcomes. However, for fostering students' agentic engagement, controlling teaching is not a good strategy in general. Finally, teachers might want to adopt a relatedness motivating teaching style, which is not only conducive to students' agentic engagement, but can play a vital role in affecting their goal orientations.

## Conclusion

We also need to point out two possible limitations. First, we conducted a cross-sectional examination of the focal issue, but peoples' motivation and goal orientation are dynamic (e.g., Reeve, 2013; Matos et al., 2018; Reeve and Shin, 2020). Most of the participants in this study were still new in university, their experience of and reaction to teachers' teaching style may subject to change over time, and so are their motivational states. It is therefore meaningful to conduct longitudinal studies to examine the dynamics of the relationship among teachers' teaching style, achievement goals and agentic engagement. In addition, we only located two types of achievement goals as the mediators. However, as speculated in the discussion, the variables including need satisfaction and frustration, and the intrinsic and extrinsic motivation, as frequently examined in the SDT literature, may join force with achievement goals to influence the relationship of perceived teachers' teaching style to students' agentic engagement. Future studies involving, say, both need satisfaction and mastery-approach goals as mediators, may help to generate more insights.

Despite these limitations, the findings of the study can add new knowledge to the literature on students' agentic engagement by uncovering how perceived autonomy support, social relatedness and controlling may play a role with the mediation of master-approach and performance approach goals. To the authors' knowledge, this study can be the first to reveal the relationship of these variables informed by an SDT and AGT integrated perspective. In addition, the results also add empirical evidence to the merit of marrying the two theories in revealing the relationship between teachers' teaching style and students' motivated behaviors.

## Data Availability Statement

The raw data supporting the conclusions of this article will be made available by the authors, without undue reservation.

## Ethics Statement

The studies involving human participants were reviewed and approved by Northeast Normal University. Written informed consent for participation was not required for this study in accordance with the national legislation and the institutional requirements.

## Author Contributions

All authors listed have made a substantial, direct and intellectual contribution to the work, and approved it for publication.

## Conflict of Interest

The authors declare that the research was conducted in the absence of any commercial or financial relationships that could be construed as a potential conflict of interest.
