# Supplementary material for: University Teachers' Teaching Style and Their Students' Agentic Engagement in EFL Learning in China: A Self-Determination Theory and Achievement Goal Theory Integrated Perspective
Source: Front Psychol. 2021 Jun 10;12:704269. doi: 10.3389/fpsyg.2021.704269 (PMC8222777; doi:10.3389/fpsyg.2021.704269)
Supplement: Supplementary file 1 [file Data_Sheet_1.docx]

**APPENDIX**

**Perceived Teachers’ Teaching Style, Achievement Goals, and Agentic Engagement Questionnaire**

| **Perceived Autonomy Support items**  My English teacher encourages us to try our best during English classes  My English teacher tries to get us to learn new things so I want to learn more.  What’s most important is for us to progress ever year in our own skills  My English teacher considers it important for us to try to improve their own skills  My English teacher tries to get us keep trying even though we make mistakes  **Perceived Social Relatedness items**  Our English class has a good sense of unity  My English teacher considers it important for us to be united in the different teaching units that we develop throughout the course.  My English teacher tries to get us really “work together” as a team in English lessons.  My English teacher tries to get us ‘pull together’ during English lessons.  **Perceived Controlling items**  My English teacher is unaware of my needs, Wants, Goals, Preferences and Emotions  My English teacher offers external incentives such as scholarships, score ranking, contest awards and other kinds of awards as a way to encourage me to learn  My English teacher seldom explains the rationale for requests, rules, procedures, and uninteresting activities  My English teacher often counters and argues against students’ negative affect  My English teacher often displays impatience such as pushes us to produce a right answer or desired behavior.  **Performance-approach Goals items**  In our English class, it is important for me to do better than other students  In our English class, it is important for me to do well compared to others in this class.  In our English class, my goal is to get a better grade than most of the other students.  **Mastery-approach Goals items**  In our English class, I want to learn as much as possible.  In our English class, it is important for me to understand the content of this course as thoroughly as possible.  In our English class, I desire to completely master the material presented.  **Agentic Engagement items**  In our English class, I let my teacher know what I need and want.  In our English class, I let my teacher know what I am interested in.  During English class, I express my preferences and opinions.  During English class, I ask questions to help me learn.  In our English class, when I need something in this class, I’ll ask the teacher for it. |
| --- |
